# The Midwives of the Future

**Published:** 1902-07-26

**Authors:** 


### THE MIDWIVES OF THE FUTURE.

THE passage of the Midwives Bill raises an interesting question as to what will be the status of the midwives of the future. There can be but little doubt that a good deal of confusion has been introduced into the whole question by the regrettable persistence with which some medical practitioners have endeavoured to impose upon midwives the new and previously unheard-of name "midwifery nurses." This has led some of our legislators to regard the terms "trained midwife" and "trained nurse" as equivalent; and a very reasonable objection was raised, that, whereas the very object of the Bill was to ensure that all midwives should be trained, many villages were too small and too poor to support a trained nurse, and that therefore hardship would result. The objection, however, falls to the ground when one recognises how great is the difference between a nurse who has undergone an expensive training, occupying three, and in many cases four, years of her life, and a midwife who can get the whole training that is required in the course of three months. The trained nurse is one thing—generally an expensive person unless her services are given on a charitable basis—the midwife is quite another. She is a woman who attends women in labour. But she may do fifty other things besides. She probably is a married woman and looks after her own house, or she is a seamstress, or she adds to her livelihood in other ways; but in addition to all these she is ready to "go out," as occasion may require, to attend confinements in her own neighbourhood. What the new Bill does is to lay it down that if a woman chooses to add to her income in this way she shall have undergone such a modicum of training or teaching as shall prevent her from perpetuating the evil and dirty practices which have been so common a cause of mortality in the past. That is all.

Incidentally, however, it is worth noting that the introduction of the clause penalising the practice of midwifery by untrained persons must very definitely affect the standard of knowledge which the Midwives' Board will be able to demand of the candidates for registration. So long as the Bill was a measure dealing merely with the title of midwife, it was open to the Midwives' Board to run up the standard of the examination to such an extent as to make midwifery a close and somewhat high-class profession. The extension of the measure, however, puts a very different face upon the matter. The Bill, as introduced, did not touch the village midwife, the handy woman who for a few shillings "goes out" to confinements, but when not wanted employs herself in other ways. Practically the village life was untouched.

But now all is altered. The Bill is no longer one for the legalisation of high-class midwifery as a career for educated women, but one for compelling the low-class midwives to obtain at least a minimum of training; and if the Midwives' Board should be so ill-advised as to set up a high standard, and to increase the length and consequently the expense of the training, there can be no doubt that the evils prophesied by the Home Secretary and the Lord Chancellor will come to pass. It was maintained by them that in the villages the Bill would affect the poorer classes in a very harsh manner, and would cause a great deal of suffering by interfering with the work of women who, in the words of Lord Portsmouth who took the same view, "were not trained, but came in to assist from time to time." Evidently no such woman should be debarred from so acting in an emergency, but if she does it habitually and for gain she may fairly be called upon to obtain some knowledge how to do the work she undertakes, and for the performance of which she obtains payment.

If the Bill should be worked with judgment the result must be in a few years' time to make it worth the while of every village midwife to qualify herself to do her work. But the attainment of this end, which is the very object of the Bill, will depend upon the "qualification" not being too high and too costly to be attained by a very ordinary sort of person. It has to be recognised that the duty of the Board is purely and simply to maintain such a reasonable minimum of elementary knowledge as shall be accessible by the village midwives. Attendance upon difficult and abnormal cases must still fall into the hands of qualified medical practitioners, and if women wish to do this sort of practice the medical profession is now as open to them as it is to their brothers. But the vast majority of childbirths will always, let us hope, be normal cases, and for attendance on such cases a very moderate amount of knowledge is required. Up to a certain point such knowledge is essential, beyond that point it is unnecessary; and what the Midwives' Board will have to do will be with care and judgment to draw the line, and, while ensuring that the midwives of the future shall not be ignorant of essentials, to take care that no such demand for non-essential knowledge shall be set up as shall prevent the ordinary village midwife attaining to the required standard, and being, as of old, at the call of her neighbours whenever occasion shall arise.

